# An empirical investigation of acceptance, adoption and the use of E-agriculture in Nigeria

**DOI:** 10.1016/j.heliyon.2021.e07588

**Published:** 2021-07-16

**Authors:** Ibukun Eweoya, Senanu R. Okuboyejo, Oluwole A. Odetunmibi, Babafemi O. Odusote

**Affiliations:** a Department of Computer and Information Sciences, Covenant University, Ota, Nigeria; b Department of Mathematics, Covenant University, Ota, Nigeria

**Keywords:** E-agriculture, Factor analysis, Path Co-efficient, Structural Equation Modeling

## Abstract

Agriculture is one of the major forces to reckon with in the employment rate and overall economy of any nation. E-agriculture is not yet fully known to all farmers in Nigeria, hence affecting adversely production and the overall business chain. The acceptance and adoption of e-agriculture can make life better and advance the economy faster. This work investigated the acceptance of e-agriculture together with its adoption in Nigeria using questionnaires for data collection. This study seeks to discover to which extent e-agriculture is adopted by diverse categories of people with basic interest on the direct determinants of usage intention and behavior; direct determinant of user behavior, and impact moderators. The Unified Theory of Acceptance and Use of Technology model was adopted and SmartPLS 3.0 was used for the analysis of the collected data. The study establishes that performance expectancy, effort expectancy, social influence and habit were discovered as variables that have significant effect on behavioral intention for the acceptance and adoption of e-agriculture while performance expectancy was discovered to be the most significant factor that influences the usage of e-agriculture in Nigeria. It is recommended that new factors like, quality of service, privacy concerns, and enhanced farmer support can be added as new factors in future works.

## 1 Introduction

Internet advent has transformed how businesses are done, leading to terms like e-learning, e-government, e-banking, and e-commerce more recently e-agriculture (Oni et al., 2017). The nomenclature expresses the usage of internet to revolutionize businesses. E-Agriculture is defined as the design, development, conceptualization, application and evaluation of innovative ways to apply emerging Information and Communication Technologies in the remote environment, focused on agriculture (Adeyemo, 2013). Agriculture may be one of the most critical sectors in the Nigerian economy, as it is estimated to engage nearly 70% of Nigerian's labor force and contribute more than 40% of the gross domestic product (Koyenikan, 2008; FMARD, 2015). It is also projected to be the main or primary source of income to almost 2.5 billion people in the developing world (Koyenikan, 2008).

Agriculture has gone through several revolutions under different governments in Nigeria; ranging from operation feed the nation, structural adjustment programme agriculture, to green revolution. The most recent is the Agricultural Transformation Agenda (ATA) (FMARD, 2015) in which grassroots farmers got mobile phones free of charge to encourage e-agriculture. This gesture was never enjoyed by farmers until around 2015. Although, the term e-agriculture was alien to the farmers; but for its convenience, it is being embraced because fertilizers and implements are delivered via e-agriculture. To support the farmers, the National Information Technology Development Agency (NITDA) has an e-portal that handles e-agriculture but most farmers are unaware, let alone taking advantage of it for national economic advancement (NITDA, 2015).

In sub-Sahara Africa, it is estimated that those who live below one US dollar ($1) per day are more than half of the population, indicating some 115 million people (Adeyemo, 2013). Also, 12 out of 15-member countries ranked among the world's least developed countries. Such desperate circumstances inevitably set limits on the citizens. The adoption of e-agriculture can change the story for the better, particularly in Nigeria. A huge volume of uneducated and peasant farming population is being orientated to embrace technology in order to expanded regional market or the opportunities that might result from scientific knowledge or a freer flow of goods and capital globally. But these same difficulties of economic isolation and constrained opportunity have driven many nations like India, Nigeria, Brazil to adopt technology (e-agriculture inclusive). The countries do use weather, breeding, market forecasts and strategies as ways to overcome domestic weaknesses such as infrastructure deficit, pest troubles, ignorance, limited communication and transportation links with the outside world.

Adoption of technology in agriculture is a pointer to enhanced income, poverty reduction, better national nutrition and health, reduced food price, and employment generation. Furthermore, the ability of a farmer to obtain relevant and current information, process and use such to adoption of a new technology depends on the farmers’ educational level (Lavison, 2013; Mwangi and Kariuki, 2015). The productive green revolution of the Asian Tigers was made possible due to adoption of improved technologies. Refusal to adopt e-agriculture is a pointer to social-economic troubles, deprivation, and degradation (Mwangi and Kariuki, 2015; Jain et al., 2009). Grassroots farmers experience rainfall that was never expected or planned for, due to ignorance of weather forecasts, infertile land, poor or lack of infrastructure. Commonly, information about issues such as irrigation, agricultural input sourcing and products marketing, credit facility availability, and extension services are barely readily accessible to many farmers (Muzari et al., 2012). E-agriculture adoption is a technological solution to overcome most these aforementioned challenges.

For many decades before year 2000, e-agriculture was being adopted at a low pace and the required aspects of adoption were barely known to many farmers (Simtowe et al., 2011; Mwangi and Kariuki, 2015). Social networks and learning are key factors that determine the adoption of any new technology (Uaiene, 2009; Oni et al., 2017). These factors have been categorized through studies into other several classifications. Armstrong and Diepeveen (2008) divided the major factors that determine the level of adoption of agricultural technology into institutional, economic and social factors. Their work revealed a Farmer Decision Support Framework (FDSF) developed to aid growers in decision making. The e-agriculture platform galvanizes cropping information from experts (research papers, websites, advisers, private consultants, and government) in western Australian agriculture sector. It was challenging to evenly distribute farming materials (implements, chemicals, and fertilizers) to all farmers and this has been complemented by information websites of breeding, seed companies, government extension websites, agriculture marketing websites and others.

Using the Internet and other technologies, the Department of Agriculture and Food of Western Australia (DAFWA), provides a website giving downloadable reports and can compare varieties of crops. The FDSF uses information collected from many sources, captured, integrated, processed, and validated through the data mining tools and put in an organized useable way to the farmers. There was a proposal of an Agricultural Information Dissemination System (AgrIDS) in Australia aimed at providing grassroots farmers expert information to boost crop productivity. It was meant to deliver a timely and effective agricultural counsel on crops via internet in form of texts and images to convey experts’ knowledge. The stakeholders of the system are the farmers, Agricultural Information System (AIS), coordinators and agriculture experts. The experts are scientists giving scientific knowledge from training and experience into the system, the coordinator is a mediator between end users (farmers), the AIS, and experts. They visits the farms to get research data (soil, crop, animals, sales, weather) information and make recommendations. The farmers are registered into the system by the coordinators for subsequent e-agriculture platform benefits and feedback (Adeyemo, 2013).

Bachu et al. (2006) presented the development and testing of an AIS based, on AgrIDS named “eSagu”. Beyond controversial extension services, the system has a robust database to support decision making, enhances correct problem diagnosis, it zooms satisfactorily for presentations understanding. Another exploit of the system is the readiness of a team of experts at a place for farmers’ attention for extension services. It conserves time and resources; it helps to also document the several success stories; provides content management and facilitates an effective feedback and support to the system to evaluate and enhance the performance.

According to National Communications Commission (NCC) as reported in Newswatch (2015); there are 146 million telecom subscribers in Nigeria. This can only make acceptance and adoption of e-agriculture better. This is a good platform to take advantage of e-agriculture, as a high percentage of farmers have mobile phones. The Ministry of Agriculture, under the transformation agenda of agriculture uses short message service (SMS) on mobile phones to distribute fertilizers and seedlings to farmers across Nigeria. This helps to curtail (if not totally prevents) corruption in the distribution chain, kicks out adulterated content, and enhances accessibility, transparency and accountability (Newswatch, 2015). The use of e-agriculture to distribute seedlings and fertilizers in Nigeria affords a timely planting and an appreciable increase in output, boosting the GDP. It is noteworthy that Nigerian farmers are tapping into e-agriculture by marketing their farm produce on websites, although mostly corporate farmers, for example universities and big farms, as at present (NITDA, 2015). Many blogs are also available attracting buyers of plantain suckers, fingerlings, eggs, and other fisheries and poultry products, thereby attracting unemployed graduates to agriculture as e-agriculture makes access to initial needs easier.

This work empirically investigates the adoption and acceptance of e-agriculture in Nigeria by revealing variables that may enhance the adoption and the use of e-agriculture thereby increasing production and boost economy of the nation.

The work reveals that Facilitating Conditions play a major role in the adoption of e-agriculture. In practice, elderly people runs from e-technologies like e-agriculture, but much encouragement and interesting orientation must be involved for enhanced adoption as supported by this work.

## 2 Methodology

Models that have been used to investigate acceptance or adoption of electronic transactions like e-learning, e-government, and e-banking include Technology Acceptance Model (TAM), Diffusion of Innovations (DOI) and a host of others (Chigona and Licker, 2006; Hanudin, 2007). However, TAM has recommended the need for the expansion of the space of theoretical mechanisms (Bagozzi, 2007; Benbasat and Barki, 2007; Venkatesh et al., 2007). The Unified Theory of Acceptance and Use of Technology (UTAUT) was propounded to investigate the effect of technology on user behavior, and user adoption of an information technology (Venkatesh et al., 2007; Akinbode al., 2018).

UTAUT predicts user adoption of an information technology product; it integrates eight prominent user acceptance and adoption theories (Venkatesh et al., 2003, 2012), including the Theory of Reasoned Action (TRA), Technology Acceptance Model (TAM), a model combining the TAM and the Theory of Planned Behavior (TPB), Innovation Diffusion Theory (IDT), the model of PC utilization (MPCU) and Social cognitive theory (SCT). It has been widely used as the theoretical basis of several research works on adoption and acceptance of technology as it vividly explains the user behavior in information system implementation (Venkatesh et al., 2011). Using empirical analysis method, it was discovered that Effort Expectancy (EE), social influence (SI) and performance expectancy (PE) and conducive environments (CE) are the key factors that determine user adoption (Venkatesh et al., 2012).

## 3 Sample size

Questionnaires were administered randomly to 199 major farmers that are perceived to embrace e-agriculture across Nigeria using convenient sampling plan representing an average of minimum of five farmers per state of the 36 state and 33 questionnaires for each of the six geo-political zone in the country. The entire questionnaire requested all respondents to fill their opinion based on Likert-scale of 5 (strongly agree) to 4 (agree) to 3(undecided) to 2 (disagree) and 1 (strongly disagree).

## 4 Theoretical background

The model that vividly describes the adoption and acceptance of technology is UTAUT, hence the proposed model for this study is the UTAUT and in further relation to the consumer context an extension is made to UTAUT, because level of individual technological know-how is one of the best way of IS research (Venkatesh et al., 2007; Akinbode al., 2018) and they consist of the following constructs: Facilitating Conditions (FC) Performance Expectancy (PE), Behavioral Intention (BI), Effort Expectancy (EE) and Social Influence (SI).

**Performance Expectancy (PE)** has been defined as the extent to which a user believes that using the system will enable him or her to achieve enhanced job performance. Therefore, there is a positive association between PE and the BI of the users (consumers) in responding to e-agriculture. This could be moderated by age and gender as stronger effect would be on men and most especially men who are young (Venkatesh et al., 2011).

**Effort Expectancy (EE)** has been defined as the rate of simplicity and ease of use, associated with the usability of a given system. Therefore, there is a direct positive impact of EE on the BI of users (consumers) in responding to e-agriculture. This could be moderated by gender, age and experience, in a way that stronger effect would be felt by women, especially younger women and at early stage of experience (Venkatesh et al., 2011).

**Social Influence (SI):** This has been defined as the extent to which an individual’ ability is perceived by others to be able or expected to use a system. Therefore, SI has a direct positive impact on the BI of users (consumers) in responding to e-agriculture. This could be moderated by gender, age, and experience, such that the stronger effect would be felt by women, especially older women at early stage of experience (Venkatesh et al., 2011).

**Behavioral Intention (BI):** This can be described a conscious and deliberate intention of individuals to engage in a behavior, where with increasing experience and exposure, the individuals has growing opportunities to reinforce their habit due to the fact that there is more time to encounter the cues and perform the associated behavior. The effect of BI on technology use will decrease as experience increases (Ayo et al., 2016).

**Facilitating Condition (FC)** has been defined as the extent to which an individual believes that organizational and technical infrastructure exists to support and facilitate the use of a particular system, by an individual (Venkatesh et al., 2007). Therefore, FC will have a direct positive impact on the behavioral intention of users (consumers) in responding to e-agriculture.

From the above analysis, we have deduced the following hypotheses:

***H1:*** *PE has a direct positive influence on the BI of stakeholders in responding to e-agriculture.*
***H2:*** *EE has a direct positive influence on the BI of stakeholders in responding to e-agriculture.*
***H3:*** *SI has direct positive influence on the BI of stakeholders in responding to e-agriculture.*
***H4:*** *FC has no direct positive influence on the BI of stakeholders in responding to e-agriculture.*

**Hedonic Motivation (HM)** is the pleasure gotten as a result of using a technology and it has been established that it plays a major role in determination of the use and acceptance of technology. It has a conceptualized meaning “perceived enjoyment”. It has been found to have a direct positive impact on the application and acceptance of technology. From the perspective of the user (consumer), HM is an important determinant of technology acceptance and adoption. It is therefore added to this model to predict the consumer's BI to use the technology. Therefore, HM has a direct positive impact on the BI of users (consumers) in responding to e-agriculture, and it also has a direct positive influence on the use behavior.

***H5:*** *Hedonic motivation has a* positive direct influence *on the behavioral intention of consumers in responding to e-agriculture.*

**Price Value (PV)/Cost** can be described as the consumers' cognitive tradeoff between the perceived benefits of use and the monetary cost of usage of the particular system. It focuses on the monetary value that is incurred upon using a certain system or technology, the PV of consumers responding to e-agriculture. Therefore the cost and price value of accepting and adopting a certain technology has a significant impact in consumers’ BI to use and adopt the system or technology. In a situation where the advantage of using a technology are thought to be much more than the cost in terms of monetary value then, it is concluded that such PV has a positive influence on intention to use and vice versa. Therefore, PV is a predictor of BI of consumers to use and accept a given technology.

***H6:*** *PV has a direct positive impact on the BI of consumers in responding to e-agriculture.*

**Experience and Habit (EH)** has been defined as the extent to which individuals tend to act and behave automatically as a result of learning habit with automaticity (Limayem et al., 2007). Although conceptualized rather similarly, habit has been operationalized into two viewpoints distinctively: First is viewing habit as prior behavior; while the second is viewing it as the extent to which an individual believes the behavior to be automatic (Venkatesh et al., 2011). Once activated, intentions and attitudes will guide behavior without any serious need for conscious mental activities, such as retrieval or belief formation. Therefore, habit will have a direct positive impact on the BI of consumers in responding to e-agriculture.

***H7:*** *Experience and Habit has a direct positive influence on the consumers' BI in responding to SMS forecasts. This relationship would be coordinated by gender, age and experience.*

**Figure 1:**
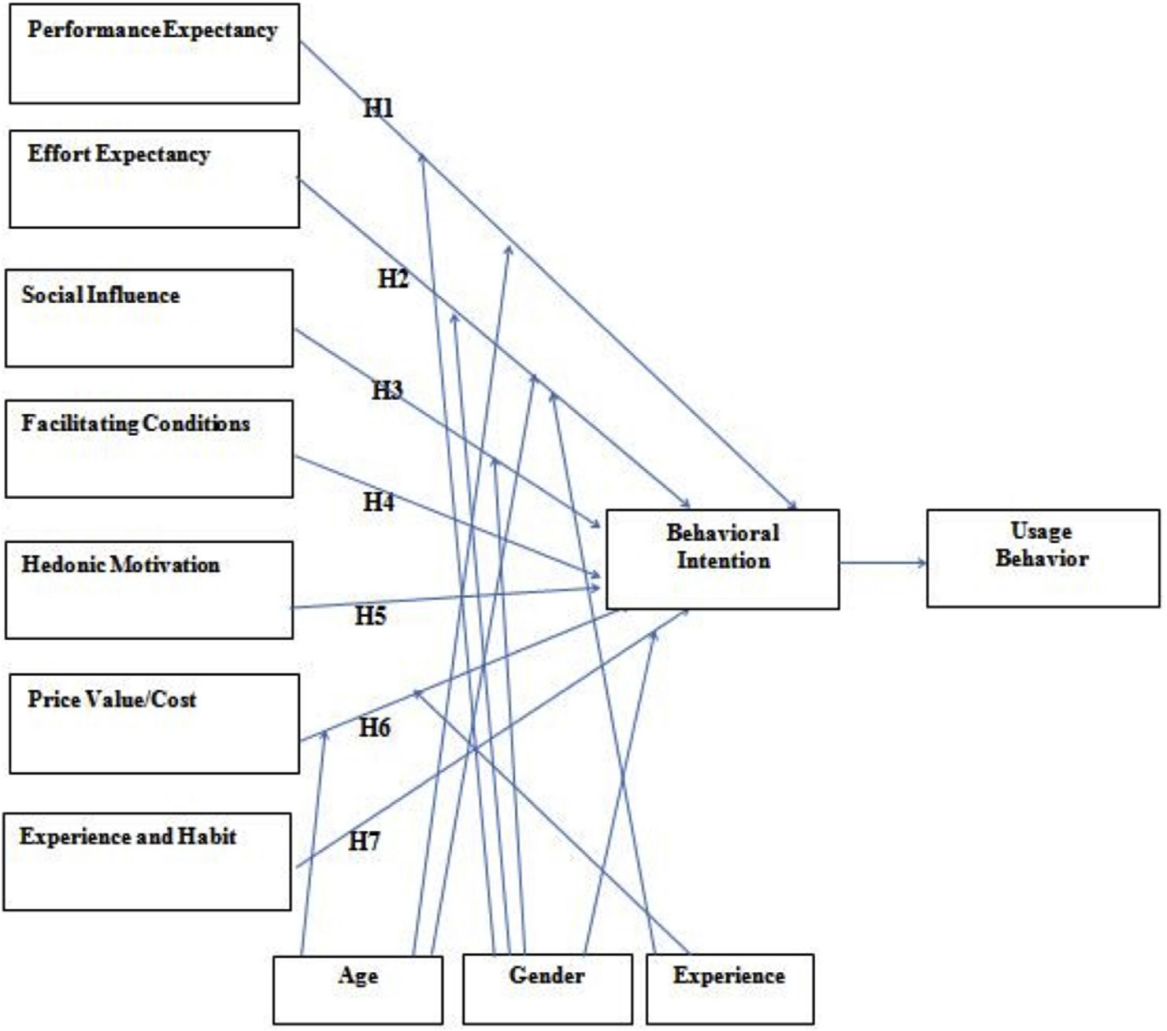
The research model.

These theories have been put together to ensure a valid research is conducted and an accurate proposal is being obtained on the effect of e-agriculture on consumer behavior. Figure 1 highlights the proposed research model which shows the relationship between all the constructs.

This work is a survey research and questionnaires were administered to collect data. It seeks to answer questions about the earlier above-mentioned constructs (variables) to be studied for adoption, acceptance and use of e-agriculture namely: FC, EE, PE, HM, SI, EH, BI, and use. Thirty-four (34) sub-questions were used collectively.

## 5 Problem statement

From meteorological services, to extension services, sale of farm produce and animal products are supposed to be enjoyed by farmers leveraging on e-agriculture; thereby maximizing profit and reducing stress. However, in Nigeria, farmers' acceptance and adoption of e-agriculture is at lower ebb (Koyenikan, 2008). Therefore, this work employs the UTAUT model to predict the acceptance and adoption of e-agriculture in Nigeria, based on the model's investigative instruments which are: Facilitating conditions, effort expectancy, social influence, behavioral intention, performance expectancy, hedonic motivation, experience or habit. The relationships of the above constructs with respect to acceptance and adoption of e-agriculture are as explained in the result presentation and justification sections.

## 6 Data analyses

The Structural Equation Modeling (SEM) was adopted for data analysis; the Confirmatory Factor Analysis (CFA) and Reliability Assessment of the measurement model were executed, and Partial Least Square (PLS) for the hypotheses testing. Partial Least Square 3.0 software (SmartPLS 3) was used to analyze the data gathered in order to validate the reliability of the variables (Ringle et al., 2015).

PLS allows for modeling of indicators as either formative or reflective on their latent construct. Furthermore, PLS has a power of prediction, as it gives room for the construction of silent variables by indicators to second order construct. The features above make PLS one of the methods that are appropriate for this research as the study is prediction-oriented.

## 7 Presentation of result

I. Demographic profile of respondents

This section shows the demographic profile and distribution of the respondents in terms of sex (gender), age, year of expertise, academic qualification, department and class.

The result from Table 1 shows that 174(87.44%) of the respondents were male while 25(12.56%) of the respondents were female. The purpose of the gender analysis was to determine whether both gender were put into consideration when the questionnaires were distributed.

**Table 1:** Sex of the respondents.

Gender	Frequency	Percentage
Male	174	87.44
Female	25	12.56

Total	199	100.00

Following from Table 2, the result shows that many of the respondents involved in this research are within the age bracket of 40–49 (31.66% of the respondents), followed by age bracket 50–59 years (22.11% of the respondents). Furthermore, 42(21.11%) of the respondents are within 30–39 years of age, 38 of the respondents falls within the age 60–69, 8 of the respondent falls within the age 20–29 while just 4 are within 70 and above. The result is an indication that the entire working class age brackets were captured in the research.

**Table 2:** Age of the respondents.

Age Group	Frequency	Percentage
20–29	8	4.02
30–39	42	21.11
40–49	63	31.66
50–59	44	22.11
60–69	38	19.09
70 & above	4	2.01

Total	199	100.00

Investigating the years of experience of the respondents, the result presented in Table 3 reveals that, 67 of the respondents have between 6-10 years of experience accounting for 33.67% of the respondents. 48(24.12%) of the respondents falls within 11–15 years of experience, 36(18.09%) are within 1–5 years of experience, while 32(16.08%) are within 0–5 years of experience. This suggests that the research relatively captured a wide range of years of experience.

II. Measurement model assessment

**Table 3:** Years of experience.

Years	Frequency	Percentage
1–5	36	18.09
6–10	67	33.67
11–15	48	24.12
16–20	32	16.08
21 & above	17	8.54

Total	199	100.00

The model validity and reliability were investigated using Composite Reliability, Cronbach's Alpha, Average Variance Extracted (AVE) and Confirmatory Factor Analysis (CFA). It has been stated that, to be retained for the next analysis, an item should have a minimum of 0.707 loading on its theoretical assigned latent construct. Twelve measurement items (PE1, EE4, SI2, FC2, FC3, HM3, PV1, HT2, HT3, HT4, BI1, B13) have factor loadings below the minimum value, and were exempted from subsequent analysis while the factor analysis results of the instrument that are refined are presented in Table 4, where all the constructs obey the minimum limits of 0.7. Also, Table 5 represents the internal consistency of the model and discriminant validity of the model were assessed using Cronbach's Alpha; many constructs including PE, EE, SI, HM, PV, HT, and FC satisfied the internal consistence condition of minimum 0.7 value with smart PLS 3.0. All constructs have a minimum of 0.5 value as stipulated for AVEs; also, PE, and HM are close to the minimum value, and retained.

**Table 4:** Evidence of unidimensionality of multiple-item sub-constructs.

Construct	Item	Factor Loading	Composite Reliability	Cronbach's Alpha	AVE	Path Co-efficient
Performance Expectancy	PE2	0.665	0.715	0.507	0.419	0.0243
	PE3	0.756				
	PE4	0.788				
Effort Expectancy	EE1	0.958	0.786	0.753	0.519	-0.097
	EE2	0.705				
	EE3	0.788				
	EE4	0.840				
Social Influence	SI1	0.708	0.620	0.542	0.454	-0.013
	SI3	0.928				
Facilitating Conditions	FC1	0.686	0.780	0.721	0.481	0.152
	FC4	0.915				
Hedonic Motivation	HM1	0.681	0.694	0.417	0.448	0.146
	HM2	0.840				
Price Value	PV2	0.735	0.702	0.357	0.454	0.446
	PV3	0.796				
Habit	HT1	0.839	0.775	0.662	0.468	-0.061

**Table 5:** Discriminant validity.

	AGE	BI	EE	EXP	FC	GEN	HM	HT	PE	PV	SI	USE
AGE	1.000											
BI	0.009	0.632										
EE	0.045	0.180	0.721									
EXP	0.213	0.020	-0.132	1.000								
FC	0.055	0.452	0.250	0.096	0.694							
GEN	-0.27	-0.039	0.072	-0.483	-0.089	1.000						
HM	0.041	0.366	0.220	-0.029	0.495	0.040	0.67					
HT	-0.04	0.591	0.394	-0.039	0.454	0.009	0.45	0.68				
PE	-0.04	0.453	0.145	-0.002	0.399	0.038	0.46	0.37	0.65			
PV	0.182	0.352	0.254	-0.014	0.391	-0.045	0.38	0.34	0.18	0.67		
SI	0.037	0.297	-0.154	0.104	0.567	0.015	0.32	0.20	0.37	0.31	0.67	
USE	-0.084	0.181	0.177	-0.072	0.025	0.029	0.023	0.065	0.116	0.113	0.023	1.000

Discriminant validity test was done so that we could check and test for the relationship that exists among the set of the variables that were used for this research and the results are presented in Table 5. In situation where square root transformation and log transformation moved skewed distributions closer to normality, a log transformation is required in order to normalize the distribution.

## 8 Interpretation of results

The result from Table 4 shows that performance expectancy has a direct positive influence on the behavioral intention of stakeholders in responding to e-agriculture since the path coefficient value (0.0243) is less than 0.05. This is an indication that respondents’ feels acceptance, adoption and the usage of e-agriculture will be determined by the performance expectancy of e-agriculture. The path co-efficient value for effort expectancy (-0.097) shows that it has a direct positive influence on the behavioral intention of stakeholders in responding to e-agriculture since is less than 0.05 and therefore will influence the acceptance, adoption and the usage of e-agriculture. Social Influence variable is believed to have positive influence on the acceptance, adoption and the usage of e-agriculture since the path co-efficient value that was obtained from the data analysis and presented in Table 4 is -0.097. All the result presented above are in tandem with the results in Hanudin (2007)**,** Chigona and Licker (2006), Bagozzi (2007)**.**

The result presented in Table 4 also shows that facilitating conditions and hedonic motivation may not influence the acceptance, adoption and the usage of e-agriculture since their path coefficient value of 0.152 and 0.146 are less than 0.05. These are in agreement with the results in Venkatesh et al. (2011, 2012); and Limayem et al. (2007). The path coefficient value for price value (0.446) suggests that the acceptance, adoption and the usage of e-agriculture will not be influence by price value. It was discovered from the analysis that habit has a direct positive impact on the behavioral intention of the respondents and therefore will influence the acceptance, adoption and the usage of e-agriculture since its path coefficient value of -0.061 is less than 0.05.

Discriminant validity test result for all the variables under investigation are presented in Table 5. The result shows that none of the variables are highly correlated and highly related which address the issue of ‘*Multi co-linearity’*. This made the variables that were used in this research work relevant and reliable for the work.

**Figure 2:**
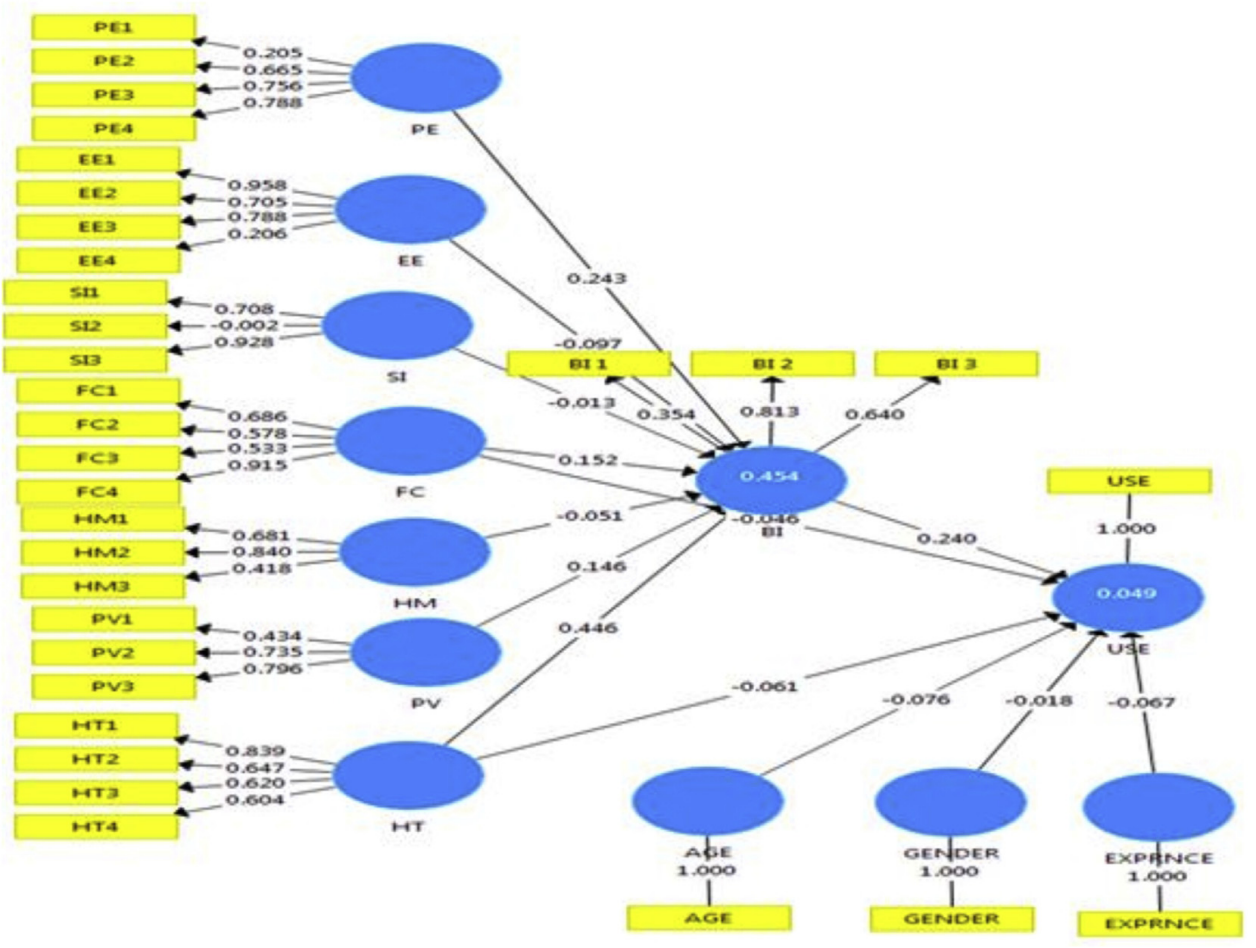
Structural Equation Model (SEM) with path analysis.

Figure 2 presents the structural models for the variables under consideration. The figure shows the relationship that exist between the independent and dependent constructs with path co-efficient values. The significance of the moderated effects in our model suggests that managers can use a market segmentation strategy to facilitate consumer technology use. Our results show that different cohorts of stakeholders attach different weights to various factors that influence their technology use, which can potentially be attributed to the differential learning abilities and social roles across age, experience, and gender.

We found that when people of advanced age are in the early stages of using a particular technology, they rely more on external resources to facilitate their continued use of the technology. This suggests that on-going facilitations designed for older people should be provided by IT application vendors if they want to keep this group of consumers on track. For instance, customer help through a call center, instant messaging services, or a consumer community can take special care of older people users who are new to IT applications. Second, we found that younger men in the early stages of experience are motivated more by the hedonic benefits gained from using a technology. This implies that hedonic applications of the technology that are interesting to younger folks with special promotions to attract younger people new to the technology.

### 8.1 Justification of research results

Thus, when the goal is to facilitate changes in consumers' habitual usage as in the case of launching a new technology, more resources may need to be targeted at elderly people with significant experience because they may have great difficulty in changing their habits. In contrast, when IT application providers want to maintain consumers’ habitual use, more attention should be paid to younger ones as they are most sensitive to changes in the environment.

## 9 Conclusions

Agriculture is as important as the existence of man. The needs of human beings have changed dynamically across decades, requiring a change in agricultural production practices. Technology cannot be ignored in the feeding of an exponentially increasing world population, and particularly in Nigeria, therefore the need for the acceptance and adoption of e-agriculture. Our results indicate divergences and convergences with findings that have been made before; hypothesis 1, 2, 3, and 7 were sustained that is, performance expectancy, effort expectancy, social influence and habit were found to have significant effect on the acceptance, adoption and the usage of e-agriculture while hypotheses 4, 5 and 6 were rejected which shows that facilitating conditions, hedonic motivation and price value do not significantly influence the acceptance, adoption and usage of e-agriculture.

To improve on this work, new factors like, quality of service, privacy concerns, and enhanced farmer support can be added as new factors to the UTAUT. The inclusion of these factors can lead to more discoveries on the acceptance, adoption and usage of e-agriculture in Nigeria.

## Declarations

### Author contribution statement

Ibukun Eweoya: Conceived and designed the experiments; Performed the experiments; Contributed reagents, materials, analysis tools or data; Wrote the paper.

Senanu R. Okuboyejo: Conceived and designed the experiments; Analyzed and interpreted the data; Contributed reagents, materials, analysis tools or data; Wrote the paper.

Oluwole A. Odetunmibi: Performed the experiments; Analyzed and interpreted the data; Contributed reagents, materials, analysis tools or data; Wrote the paper.

Babafemi O. Odusote: Performed the experiments; Contributed reagents, materials, analysis tools or data; Wrote the paper.

### Funding statement

This research did not receive any specific grant from funding agencies in the public, commercial, or not-for-profit sectors.

### Data availability statement

Data will be made available on request.

### Declaration of interests statement

The authors declare no conflict of interest.

### Additional information

No additional information is available for this paper.

## Appendix A Supplementary data

E-agriculture questionnaire E-agriculture questionnaire
