# Supplementary material for: An empirical investigation of acceptance, adoption and the use of E-agriculture in Nigeria
Source: Heliyon. 2021 Jul 16;7(7):e07588. doi: 10.1016/j.heliyon.2021.e07588 (PMC8340116; doi:10.1016/j.heliyon.2021.e07588)
Supplement: E-agriculture questionnaire [file mmc1.doc]

**E-AGRICULTURE SURVEY QUESTIONNAIRE.**

**Sir/Ma,**

This questionnaire is to gather information about usage, acceptance and adoption of e-agriculture in Nigeria. We hereby confirm the confidentiality of your information as we appreciate your time.

**E-Agriculture** involves the conceptualization, design, development, evaluation and application of innovative ways to use information and communication technologies in the rural domain, with a primary focus on agriculture**.**

**SECTION A: BIO DATA**

**Sex:**

| S/N |  | **Responses [Just tick]** |
| --- | --- | --- |
| A | Male |  |
| B | Female |  |

Age Bracket

| S/N |  | **Responses [Just tick]** |
| --- | --- | --- |
| A | 20-29 |  |
| B | 30-39 |  |
| C | 40-49 |  |
| D | 50-59 |  |
| E | 60-69 |  |
| F | 70 and above |  |

**Years of Experience**

| S/N |  | **Responses [Just tick]** |
| --- | --- | --- |
| A | 1-5years |  |
| B | 6-10years |  |
| C | 11-15year |  |
| D | 16-20years |  |
| E | 21 year and above |  |

**Geo-political zone**

| **S/N** |  | **Responses [Just tick]** |
| --- | --- | --- |
| A | South West |  |
| B | South East |  |
| C | South South |  |
| D | North Central |  |
| E | North East |  |
| F | North West |  |

**SECTION B: SURVEY ITEMS**

| ***Performance Expectancy*** | **STRONGLY AGREE** | **AGREE** | **UNDECIDED** | **DISAGREE** | **STRONGLY DISAGREE** |
| --- | --- | --- | --- | --- | --- |
| PE1. I find e- agriculture useful in my daily life. |  |  |  |  |  |
| PE2. Using e- agriculture can increase my chances of achieving things that are important to me. |  |  |  |  |  |
| PE3. Using e- agriculture can help me accomplish things more quickly. |  |  |  |  |  |
| PE4. Using e- agriculture can increase my productivity. |  |  |  |  |  |
| ***Effort Expectancy*** |  |  |  |  |  |
| EE1. Learning how to use e- agriculture is easy for me. |  |  |  |  |  |
| EE2. My interaction with e- agriculture is clear and understandable |  |  |  |  |  |
| EE3. I find e- agriculture easy to use. |  |  |  |  |  |
| EE4. It is easy for me to become skillful at using e- agriculture platforms. |  |  |  |  |  |
| ***Social Influence*** |  |  |  |  |  |
| SI1. People who are important to me think that I should use e- agriculture. |  |  |  |  |  |
| SI2. People who influence my behavior think that I should use e- agriculture. |  |  |  |  |  |
| SI3. People whose opinions that I value prefer that I use e- agriculture. |  |  |  |  |  |
| ***Facilitating Conditions*** |  |  |  |  |  |
| FC1. I have the resources necessary to use e- agriculture. |  |  |  |  |  |
| FC3. E- agriculture is compatible with other technologies I use. |  |  |  |  |  |
| FC4. I can get help from others when I have difficulties using e- agriculture platforms. |  |  |  |  |  |
| ***Hedonic Motivation*** |  |  |  |  |  |
| HM1. Using e- agriculture is fun. |  |  |  |  |  |
| HM2. Using e- agriculture is enjoyable. |  |  |  |  |  |
| HM3. Using e- agriculture is very encouraging. |  |  |  |  |  |
| ***Price Value*** |  |  |  |  |  |
| PV1. E- agriculture is reasonably priced. |  |  |  |  |  |
| PV2. E- agriculture is a good value for the money. |  |  |  |  |  |
| PV3. At the current price, e- agriculture provides a good value. |  |  |  |  |  |
| ***Habit*** |  |  |  |  |  |
| HT1. The use of e- agriculture can become a habit for me. |  |  |  |  |  |
| HT2. I can be addicted to using e- agriculture. |  |  |  |  |  |
| HT3. I must use e- agriculture. |  |  |  |  |  |
| HT4. Using e- agriculture can become natural to me. |  |  |  |  |  |
| ***Behavioral Intention*** |  |  |  |  |  |
| BI1. I intend to continue using e- agriculture in the future. |  |  |  |  |  |
| BI2. I will always try to use e- agriculture in my daily life. |  |  |  |  |  |
| BI3. I plan to continue to use e- agriculture frequently. |  |  |  |  |  |

***Use***

Please choose your usage frequency for each of the followings in relation to e-agriculture:

a) SMS ……….

b) MMS ………

c) Weather forecast alerts.……

d) E-agric. Extension service ……….

e) Browse websites ………….

f) Mobile e-mail ………………..

*Note*: Frequency ranges from “never” to “many times per day i.e MTPD.”
